# Total Osseous Calcification of the Prostate Gland

**DOI:** 10.7759/cureus.9239

**Published:** 2020-07-17

**Authors:** Filipos Kapogiannis, Konstantinos Fasoulakis, Charalampos Fragkoulis, Angelos Aggelopoulos, Charalampos Fasoulakis

**Affiliations:** 1 Urology, Hippokration General Hospital, Athens, GRC; 2 Urology, General Hospital of Athens "G. Gennimatas", Athens, GRC

**Keywords:** prostatic stones, osseous prostate calcification, bladder stones, open prostatectomy

## Abstract

Prostatic calculi are commonly seen in older men as their incidence increases with age. They are associated with prostate hypertrophy, chronic inflammation of the gland, prostate cancer, and rarely with other pathological conditions such as granulomatous diseases. Although typical small in size, they can seldom become giant and replace the entire prostate gland with only few cases reported in literature. We present one such rare case of a young male who presented to the emergency department with clinical manifestations of sepsis and no relevant past medical history. The patient was ultimately treated with open simple retropubic prostatectomy as a surgical stone extraction method.

## Introduction

Prostatic calculi correspond to calficication material developed in the vicinity of the prostate tissue and should be distinguished from calculations blocked in the prostatic urethra and are of bladder or renal origin. The latter is often a cause of obstruction to the flow of urine and a substanial contributor of lower urinary tract symptoms. They are classified into two groups according to their origin: endogenous are formed from prostatic secretions and exogenous or secondary are formed within the prostatic ducts from constituents of the urine. Prostatic parenchymal calculi are usually incidental findings on a CT scan or transrectal ultrasound because they are typically asymptomatic. We present a patient with a total replacement of his prostate gland with a giant calculus and multiple bladder stones who presented to the emergency department with signs and symptoms sepsis.

## Case presentation

**Figure 1:**
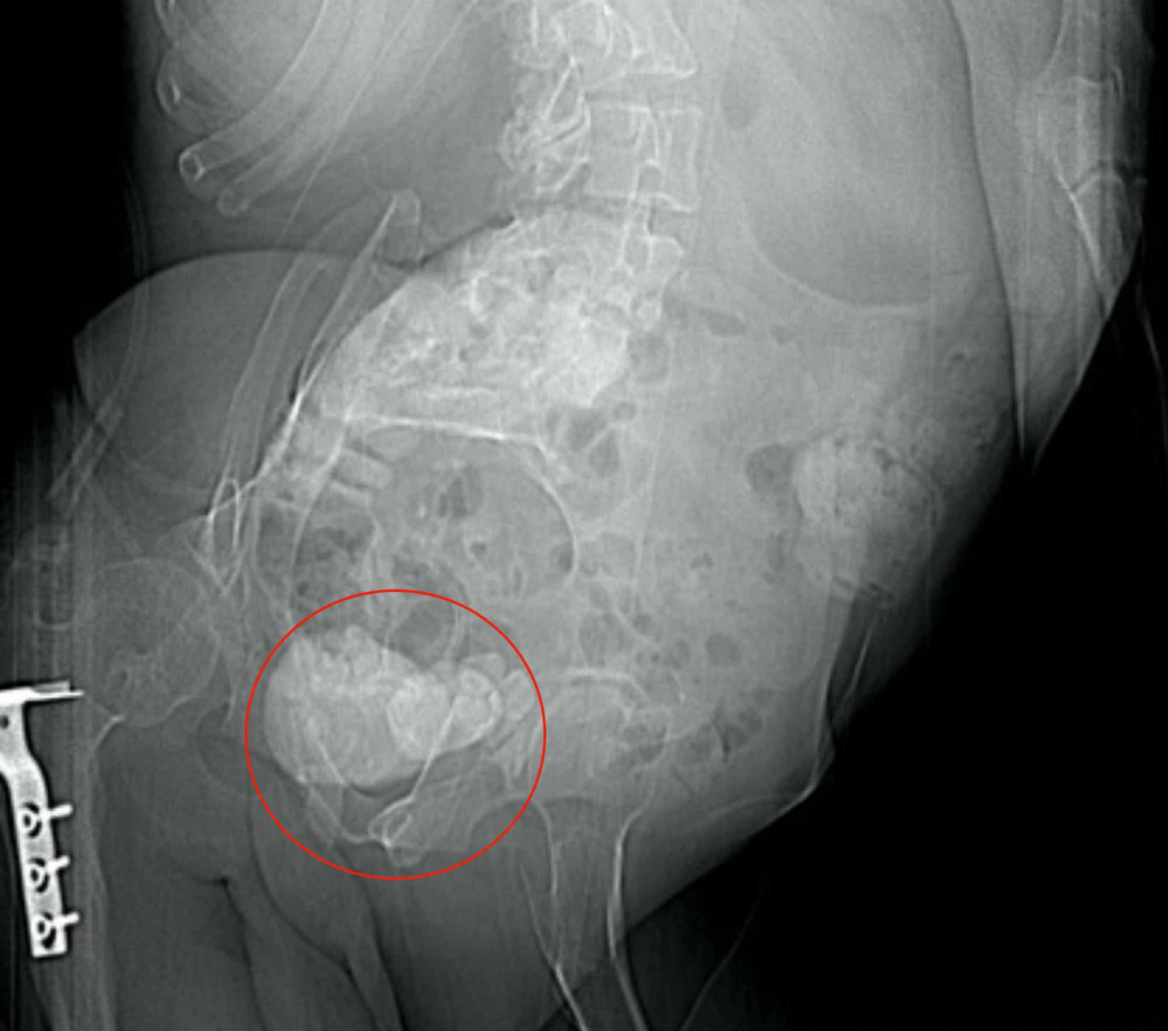
Plain X-ray KUB shows large stone burden in the urinary bladder and the prostate gland KUB: kidneys, ureters and bladder

A 44-year-old male with disabilities and prominent kyphoscoliosis presented to our “Acute and Emergency” department and reported worsening lower urinary tract symptoms along with oliguria and fever. He had a past medical history of meningocele repair and did not receive any recent medication. The patient also reported a known history of low capacity/compliance bladder based on previous urodynamic study. His blood results demonstrated marked leucocytosis at 18 x 10^9^/L, elevated C-reactive protein and acute kidney injury. Prostate-specific antigen was 0.15 ng/ml. Urinanalysis in the emergency department demonstrated microscopic haematuria, leucocytes and nitrites. Plain X-ray of kidneys, ureters and bladder (KUB) revealed gross prostatic calcification along with bladder stones (Figure 1).

**Figure 2:**
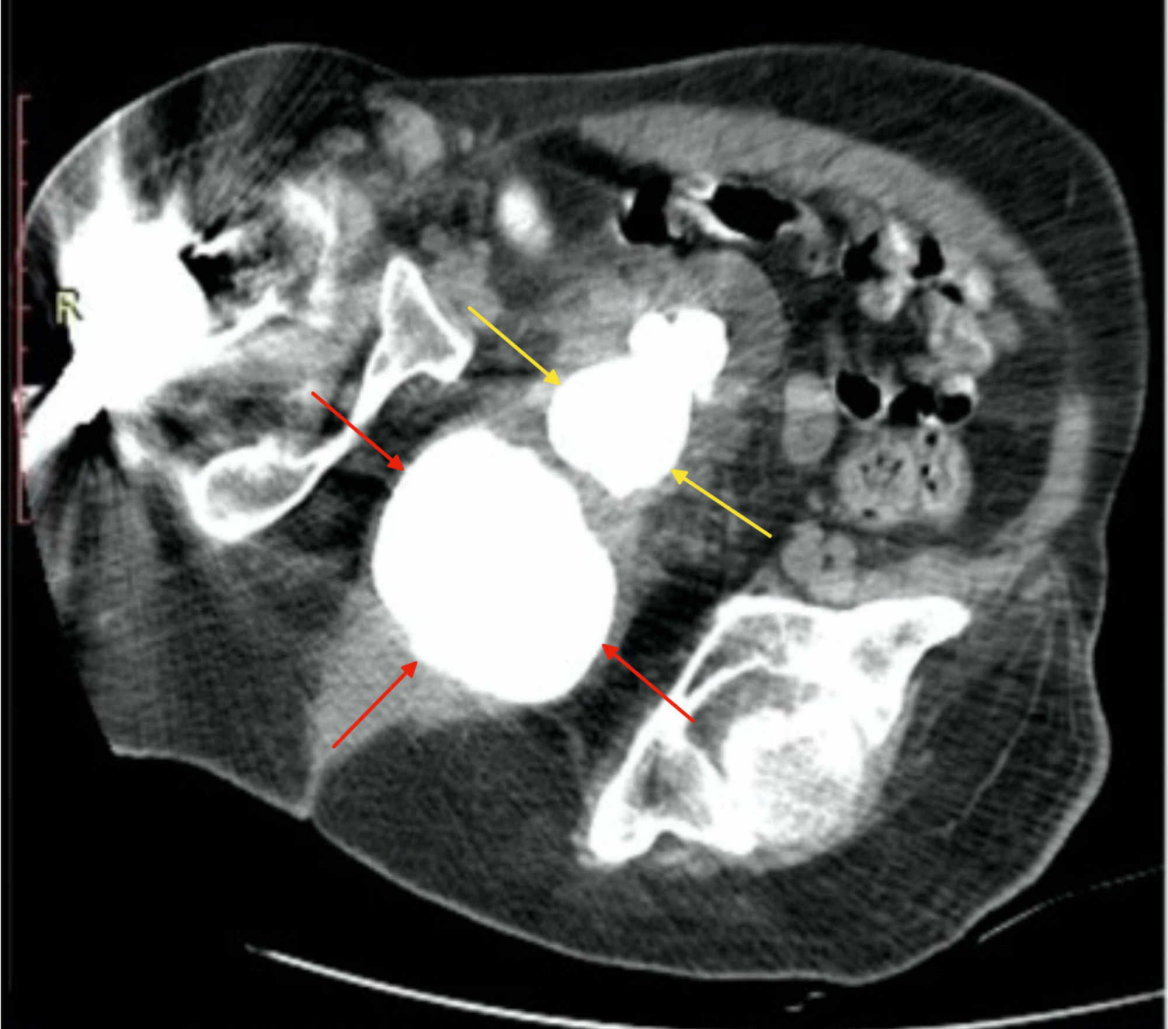
Pelvic CT reveals an osseous transformation of the prostate (red arrows) and multiple bladder stones (yellow arrows)

Upon failure of inserting both a transurethral catheter due to blockage in the prostatic urethra and a suprapubic catheter because of an empty bladder, the patient subsequently underwent imaging investigation with a non-contrast CT of the abdomen. The CT findings revealed severe symmetrical bilateral hydronephrosis and dilatation of the ureters up to the bladder level and confirmed the calcification previously seen on conventional KUB X-ray (Figure 2).

We decided to place bilateral nephrostomies after the administration of empiric, broad-spectrum antimicrobial therapy as per local guidelines and as the only solution to treat the potentially life-threatening septic condition. After the placement of bilateral nephrostomies, the patient became apyrexial, and his symptoms and blood results improved markedly. He remained inpatient for seven days being discharged with a 12Fr bilateral nephrostomy and oral antibiotic cover for two weeks as per urine culture.

One month later, the patient was subjected to an open retropubic transvesical prostatectomy (Freyer procedure). Intraoperatively, urinary bladder demonstrated excessive wall thickness and extensive trabeculation, and its lumen was occupied by multiple calculi that were retrieved with a stone forceps and extracted. On palpation, the prostate had lost its elasticity and felt of irregular periphery and stony hard. Nearly all of its parenchyma had been replaced by stone tissue. Fragmentation of the prostate calculi due to size and hardness was achieved in situ by means of using orthopaedic surgical instruments, such as jaw pliers and bone-cutting forceps.

**Figure 3:**
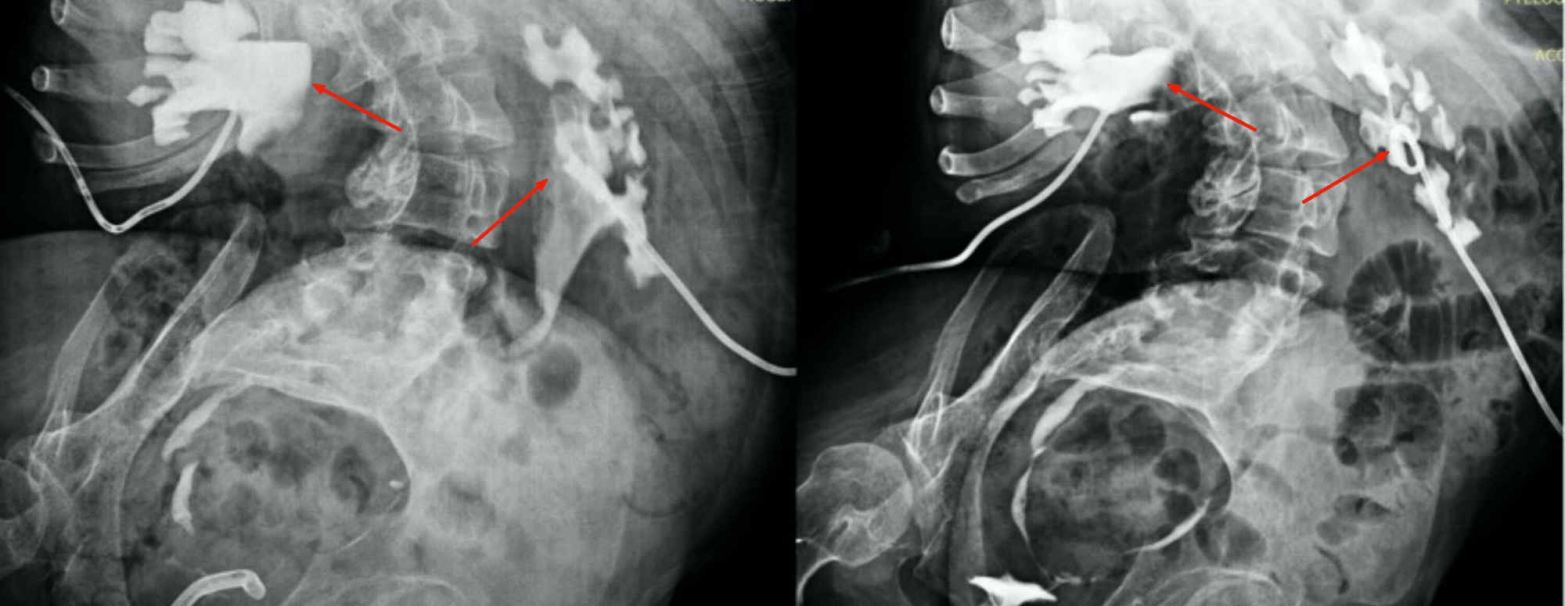
Nephrostomography of both kidneys showing mild bilateral ditalation of the ureteropelvic junction (arrows left) which resolved in a control study a few days later (arrows right)

The postoperative period was uneventful apart from mild dilatation of both collecting systems on nephrostomography due to oedema of the ureteropelvic junction which resolved partly in a control study few days later (Figure 3). The patient was discharged on the 10th day following surgery after removal of the nephrostomies and a successful trial without a transurethral catheter with minimal residual bladder urine.

**Figure 4:**
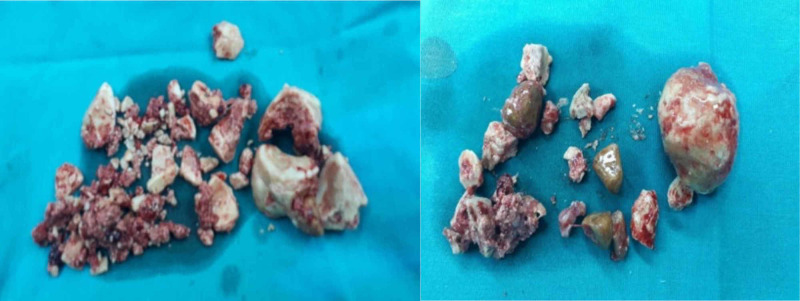
Extracted fragments of calculi from the prostate (left) and the urinary bladder (right)

Stone analysis revealed predominantly calcium oxalate and after a thorough laboratory work-up and in the absence of obvious explanation for calcium stone formation, the patient was referred to as idiopathic calcium stone former. He was advised on an oxalate-controlled diet and further individualized metabolic evaluation with the collaboration of a specialized nephrologist (Figure 4).

## Discussion

Prostatic calcification is a quite common urological condition whose clinical significance remains partly undetermined. Donatus in 1586 described first this clinical entity, followed by Pohl in 1737 [1]. Regarding the incidence of prostatic calculi, recent studies have shown they are found seldom in paediatric population, infrequently in men up to 40 years of age and commonly in middle-age men or older men unlike prostatic urethral calculi which usually occur in younger men [2-5]. True prostatic calculi are in fact a result of a vicious cycle, which starts with inflammatory congestion of prostatic acini, continues with stasis and retention of the prostatic fluid along with desquamation of acinar cells, and ends with formation of corpora amylacea and deposition of calcium salts [6].

Prostatic calcification is commonly located at the apical margin of the prostatic adenoma in the cephalad portion of the gland (pseudocapsule) but it may also be identified in the region of verumontanum and ejaculator duct. On the other hand, urethral calculi are predominantly found in the caudal portion of the periurethral prostatic tissue according to distribution in grey-scale transrectal ultrasound examination [7].

Based on their composition, prostatic calculi have been further classified as primary or endogenous calculi and secondary or exogenous calculi composed primarily of apatite and whitlockite on chemical analysis [8].

There has been an association of development of prostatic calculi with a number of causative factors or pathological conditions, such as chronic prostatitis, prostatic hyperplasia, prostatic carcinoma, radiotherapy, tuberculosis, schistosomiasis, foreign bodies, debris and lithogenic diathesis. Predisposing factors for in situ development of urethral stones include the presence of urethral diverticulum, urethral stricture, hypospadias and meatal stenosis.

Some authors have reported that prostatic calculi produce non-specific lower urinary tract symptoms or even acute urinary retention [9,10]. They may often be completely asymptomatic or may have few symptoms like burning sensation in the urethra on urination, burning sensation in the perineum and/or rectum, perineal or penile pain, frequency, urgency, diminished urinary stream and urethral discharge. Other less frequent symptoms were haematuria, dribbling or incontinence, interruption of the urinary stream and a history of having passed a stone [11,12]. The patient’s medical history and routine urological work-up will reveal the cause in the majority of the cases.

Typically symptomatic prostatic calculi do not require treatment. A number of treatment options according to location and size of the stones have been described in the past, including extracorporeal shock wave lithotripsy, endoscopic lithotripsy, open retropubic prostatolithotomy, cystotomy with bladder neck incision, perineal urethrotomy and radical prostatectomy [13].

## Conclusions

This is the first case in our centre of combined treatment of multiple bladder stones and osseous replacement of prostate gland in the same patient as simultaneous presence is very unusual. Less than 30 cases of giant prostatic calculi have been reported in the English literature and even less reports exist of virtually total osseous transformation of the prostatic tissue. Although a minimal invasive approach should be the first choice of therapy, open surgery in selected cases seems to be the only choice of treatment in order for the patient to become stone-free and to avoid the necessity of repeat intervention.
